# Dihydroartemisinin Inhibits the Proliferation of Esophageal Squamous Cell Carcinoma Partially by Targeting AKT1 and p70S6K

**DOI:** 10.3389/fphar.2020.587470

**Published:** 2020-11-20

**Authors:** Lili Zhu, Xinhuan Chen, Yanyan Zhu, Jiace Qin, Tingting Niu, Yongwei Ding, Yang Xiao, Yanan Jiang, Kangdong Liu, Jing Lu, Wanjing Yang, Yan Qiao, Ge Jin, Junfen Ma, Ziming Dong, Jimin Zhao

**Affiliations:** ^1^ Department of Pathophysiology, School of Basic Medical Sciences, Zhengzhou University, Zhengzhou, China; ^2^ Department of Pathology, The First Affiliated Hospital, School of Medicine, Zhejiang University, Hangzhou, China; ^3^ Henan Provincial Cooperative Innovation Center for Cancer Chemoprevention, Zhengzhou, China; ^4^ Department of Dermatology, The Second Affiliated Hospital, School of Medicine, Xi’an Jiaotong University, Xi’an, China; ^5^ The China-US (Henan) Hormel Cancer Institute, Zhengzhou, China; ^6^ Department of Biochemistry and Molecular Biology, School of Basic Medical Sciences, Zhengzhou University, Zhengzhou, China; ^7^ Department of Clinical Laboratory, The First Affiliated Hospital, Zhengzhou University, Zhengzhou, China; ^8^ State Key Laboratory of Esophageal Cancer Prevention and Treatment, Zhengzhou University, Zhengzhou, China

**Keywords:** esophageal squamous cell carcinoma, dihydroartemisinin, AKT1, mTOR, p70S6K, RPS6, patient-derived xenograft

## Abstract

Dihydroartemisinin (DHA), a sesquiterpene lactone with endoperoxide bridge, is one of the derivatives of artemisinin. In addition to having good antimalarial properties, DHA exhibits anticancer effects including against malignant solid tumors. However, the mechanism by which DHA inhibits the progression of esophageal cancer, especially esophageal squamous cell carcinoma (ESCC), is unclear. In this study, DHA was found to inhibit the proliferation of ESCC, and the underlying molecular mechanisms were explored. DHA inhibited ESCC cells proliferation and anchorage-independent growth. Flow cytometry analysis revealed that DHA significantly blocked cell cycle in the G1 phase. The results of human phospho-kinase array revealed that DHA downregulated the levels of p70S6K^T389^ and p70S6K^T421/S424^. Furthermore, the levels of mTOR^S2448^, p70S6K^T389^, p70S6K^T421/S424^ and RPS6^S235/S236^ were decreased after DHA treatment in KYSE30 and KYSE150 cells. We then explored the proteins targeted by DHA to inhibit the mTOR-p70S6K-RPS6 pathway. Results of the *in vitro* kinase assay revealed that DHA significantly inhibited phosphorylation of mTOR^S2448^ by binding to AKT1 and p70S6K kinases. *In vivo*, DHA inhibited the tumor growth of ESCC patient-derived xenografts and weakened p-mTOR, p-p70S6K, and p-RPS6 expression in tumor tissues. Altogether, our results indicate that DHA has antiproliferative effects in ESCC cells and can downregulate mTOR cascade pathway partially by binding to AKT1 and p70S6K. Thus, DHA has considerable potential for the prevention or treatment of ESCC.

## Introduction

Esophageal cancer, a malignant disease, ranked the seventh in morbidity and the sixth in overall mortality in the world. Its two common pathological types are esophageal squamous cell carcinoma (ESCC) and adenocarcinoma (Bray et al., 2018). ESCC is the major histological type and accounts for 90% of cases of “esophageal cancer belt” (Torre et al., 2015). The overall 5-years survival rate of esophageal cancer is less than 20% (Xiong et al., 2016), forcing both clinicians and researchers to find more effective treatments. Phytochemicals have compelling roles in cancer treatment either when used alone or as synergistically, and they also reduce costs and adverse side effects (Koh et al., 2020). In addition, phytochemicals, especially those prescribed in clinics, can be used to interfere with the progression of cancer.

*Artemisia annua*, a plant belonging to Asteraceae family, is a source of artemisinin (Dhingra et al., 2000). Artemisinin and its derivatives with an endoperoxide sesquiterpene lactone play an excellent role as antimalarial drugs. The antimalarial mechanism of artemisinin includes the formation of free radicals through the cleavage of its endoperoxide bond, which leads to the elimination of *Plasmodium* species (Ho et al., 2014). As the modification of the carboxy group to a hydroxy group improves their efficacy, dihydroartemisinin (DHA) is introduced as a new antimalarial drug (Tu, 2016).

It is noticeable, however, DHA has shown a significant inhibitory effect on a variety of cancers beyond its antimalarial effects in recent decades. For example, DHA inhibits prostate cancer by relying on Axl downregulation of JARID2/miR-7/miR-34a, leading to decreased proliferation, migration, and invasion and inhibition of tumors *in vivo* (Paccez et al., 2019). DHA shows anticancer activity and induces G_2_ cell cycle arrest and apoptosis in human ovarian cancer cells (Jiao et al., 2007). DHA also has the therapeutic potential on angiogenesis (Gao et al., 2020). These studies show that DHA has strong anticancer activities against different types of cancers with different mechanisms.

Currently, limited experimental research has investigated the potential of DHA in ESCC. DHA inhibits the growth of ESCC cells Eca109 and Ec9706 and enhances the therapeutic effect of photodynamic therapy (Li et al., 2014; Li et al., 2018). However, the specific molecular target of DHA in ESCC has not been identified. Here, we aimed to identify the inhibitory effects of DHA on ESCC cells and patient-derived xenograft (PDX) tumors *in vivo* and reveal the underlying molecular mechanism.

## Materials and Methods

### Antibodies and Reagents

The primary antibodies and their dilution ratio in western blot analysis were as follows: p-mTOR-S2448 (1:1,000, Cell Signaling Technology); mTOR (1:1,000, Cell Signaling Technology); p-p70S6K-T389 (1:1,000, Cell Signaling Technology); p-p70S6K-T421/S424 (1:1,000, Cell Signaling Technology); p70S6K (1:1,000, Cell Signaling Technology); p-S6 Ribosomal Protein -S235/S236 (1:1,000, Cell Signaling Technology); RPS6 (1:1,000,Abcam); *β*-Actin (1:1,000, Tianjin Sungene Biotech Co., Ltd., China) and GAPDH (1:1,000, Good Here, China). DHA was purchased from Tokyo Chemical Industry (Cat #D3793, Japan). MK-2206 (AKT inhibitor, Cat #A10003) was purchased from Selleckchem. LY2584702 (p70S6K inhibitor, Cat #1082949–68–5) was purchased from Med Chem Express.

### Cell Culture

KYSE30 cell line was provided by the Department of Pathophysiology, School of Basic Medical Sciences, Zhengzhou University. KYSE150 cell line was purchased from the Type Culture Collection of the Chinese Academy of Sciences (Shanghai, China). The cells were cytogenetically tested using STR technology for authentication. Cells were cultured in Roswell Park Memorial Institute 1,640 (RPMI1640) medium with 10% fetal bovine serum (FBS) and 1% penicillin-streptomycin at 37°C in a 5% CO_2_ incubator.

### Cytotoxicity Assay

Cells were seeded in 96-well plates at 8 × 10^3^ cells per well. After approximately 14 h, media were replaced with growth medium containing DHA at different doses (0, 0.3, 0.9, 2.7, 8.1, 24.3, or 72.9 µM). The cells treated with DHA were cultured for 0, 24, or 48 h and then fixed in 4% paraformaldehyde. The cells were counted using 4′,6-diamidino-2-phenylindole (DAPI) staining and an In Cell 6,000 Analyzer (GE Healthcare).

### Cell Proliferation Assay

Cells were seeded in 96-well plates at 2 × 10^3^ cells per well. After approximately 14 h, media were replaced with growth medium containing DHA at the different doses (0, 1, 5, 10, or 15 µM). The cells treated with DHA were cultured for 0, 24, 48, 72, or 96 h and were fixed in 4% paraformaldehyde. The cells were counted using DAPI staining and an In Cell 6,000 Analyzer.

### Anchorage-Independent Cell Growth Assay

Basal Medium Eagle (Sigma-Aldrich, United Kingdom) supplemented with 10% FBS, 0.5% agarose, 9% sterile water, 1% glutamine, 0.1% gentamicin and different concentrations of DHA was spread in a 6-well plate at a volume of 3 ml per well. After approximately 2 h of solidification of the medium, cells (8 × 10^3^ per well) were suspended in the top layer containing 10% FBS, 0.33% agar, 45% sterile water, 1% glutamine, 0.1% gentamicin and different concentrations of DHA (0, 1, 5, 10, or 15 µM). The cells were cultured at 37°C in a 5% CO_2_ incubator for 8 days, and colony numbers were counted using an In Cell 6,000 Analyzer.

### Western Blot Analysis

Protein samples for western blot were obtained using lysis buffer (Solarbio, Beijing, China) and identified using a BCA protein assay kit (Beyotime, Jiangsu, China). Proteins (30–50 μg) were separated in 10% sodium dodecyl sulfate-polyacrylamide gel electrophoresis and transferred to polyvinylidene difluoride membranes. The membranes were blocked with 5% non-fat milk for 1 h at room temperature and incubated with primary antibodies at 4°C overnight. Then, the membranes were incubated with horseradish peroxidase (HRP)-IgG secondary antibody (1:5,000) for 2 h at room temperature and protein bands were detected using chemiluminescence reagent by Fluor Chem E (Protein Simple, US). Densitometry analyses were performed for all western blots by ImageJ (NIH Image, Bethesda, MD).

### Cell Cycle Assay Using Flow Cytometry

After incubation in serum-free medium for 24 h in 60 mm dishes, media were replaced with complete medium (10% FBS) containing different concentrations of DHA (0, 1, 5, 10, or 15 μM) and incubated for 24 h. The cells were fixed in ice-cold 70% ethanol overnight, incubated with RNase (50 μg/ml) for 1 h, and stained with propidium iodide (50 μg/ml) for 30 min. Cell cycle distribution was measured and analyzed using FACScan flow cytometry (BD FACSCanto) and FlowJo7.6.1 software, respectively.

### Human Phospho-Kinase Array

KYSE30 and KYSE150 cells were treated with DHA (10 μM) for 12 h. Phosphorylated proteins were analyzed using the Human Phospho-Kinase Array Kit (ARY003B; R&D Systems, Inc., USA and Canada) according to the manufacturer’s instructions. The array membranes were blocked with 1.0 ml of Array Buffer one and incubated with 500 μg cell lysates. The detection antibody cocktail was added, followed by incubation with streptavidin-HRP. Signals were detected using the Chemi Reagent Mix, and array images were analyzed using ImageJ software. The negative control signal was subtracted from each spot, and values were normalized to the positive control present in each membrane. Then, the ratio of signal intensity (treated/untreated) was calculated.

### Pull-Down Assay

KYSE30 and KYSE150 cell lysates (500 μg) were incubated with DHA-Sepharose 4B (or Sepharose 4B only as a control) beads (50 μL, 50% slurry) in a reaction buffer [50 mM Tris pH 7.5, 5 mM EDTA, 150 mM NaCl, 1 mM dithiothreitol (DTT), 0.01% NP40, 2 μg/ml bovine serum albumin]. After incubation with gentle rocking overnight at 4°C, the beads were washed five times with the reaction buffer (50 mM Tris pH 7.5, 5 mM EDTA, 150 mM NaCl, 1 mM DTT, 0.01% NP40) and binding was visualized using western blot analysis.

### *In vitro* Kinase Assay

The purified mTOR protein (Cloud-Clone Corp.,Wuhan, China) was used as the substrate for an *in vitro* kinase assay with 100 ng of active AKT1 (Signalchem, Catalog No. A16-10G) or p70S6K (Signalchem, Catalog No. R21-10H). Reactions were conducted in 1X kinase buffer (25 mM Tris-HCl pH 7.5, 5 mM *β*-glycerophosphate, 2 mM DTT, 0.1 mM Na_3_VO_4_, 10 mM MgCl_2_, and 5 mM MnCl_2_) containing 200 μM ATP at 30°C for 30 min. Reactions were stopped by adding 6X protein loading buffer, and proteins were detected using western blot analysis.

### Xenograft Mouse Models

Severe combined immunodeficient (SCID), 6–8 weeks old female mice (Vital River Labs, Beijing, China) were used as the PDX mouse model following the guidelines of the Ethics Committee of Zhengzhou University (Zhengzhou, Henan, China). The two cases of ESCC in PDX animal experiments were named LEG20 and LEG24. The tumors were divided into 0.1–0.2 g fragments and inoculated into the back of SCID mice. Mice were randomly assigned to the following groups: control group, 25 mg/kg DHA-treated group, and 50 mg/kg DHA-treated group. Each mouse was administered either DHA or DMSO three times per week by intraperitoneal injection. The tumor volume and weight of each mouse were measured every 3 days. Tumor volume was calculated based on the dimensions of the individual tumor and according to the following formula: Tumor volume (mm^3^) = (length × width × width/2).

### Immunohistochemical Staining

Mice in the control group were monitored until tumors reached about 1.0 cm^3^ volume, at which time mice were euthanized and tumors were extracted. The tumor tissues were embedded in paraffin and cut into 4 μm thick sections. Slides were deparaffinized and hydrated with xylene and gradient alcohol. The tissues were treated for microwave heat-induced epitope retrieval and soaked in 0.3% hydrogen peroxide for 5 min. Subsequently, the tissues were incubated with p-mTOR (1:50), p-p70S6K (1:50), and p-RPS6 (1:200) overnight at 4°C. After incubation with HRP-conjugated secondary antibodies at 37°C for 15 min, the tissues were detected with 2,4-diaminobenzidine substrate (DAB) and counterstained with hematoxylin. All slides were scanned by using TissueFAXS and analyzed by using HistoQuest 4.0 software (both from TissueGnostics GmbH, Vienna, Austria).

### Statistical Analysis

All quantitative results are expressed as mean ± SD. Statistically significant differences were obtained by using Student’s t-test or one-way ANOVA. *p* < 0.05 was considered statistically significant.

## Results

### Dihydroartemisinin Inhibits Proliferation and Affects Cell Cycle Progression in Esophageal Squamous Cell Carcinoma Cells

**FIGURE 1:**
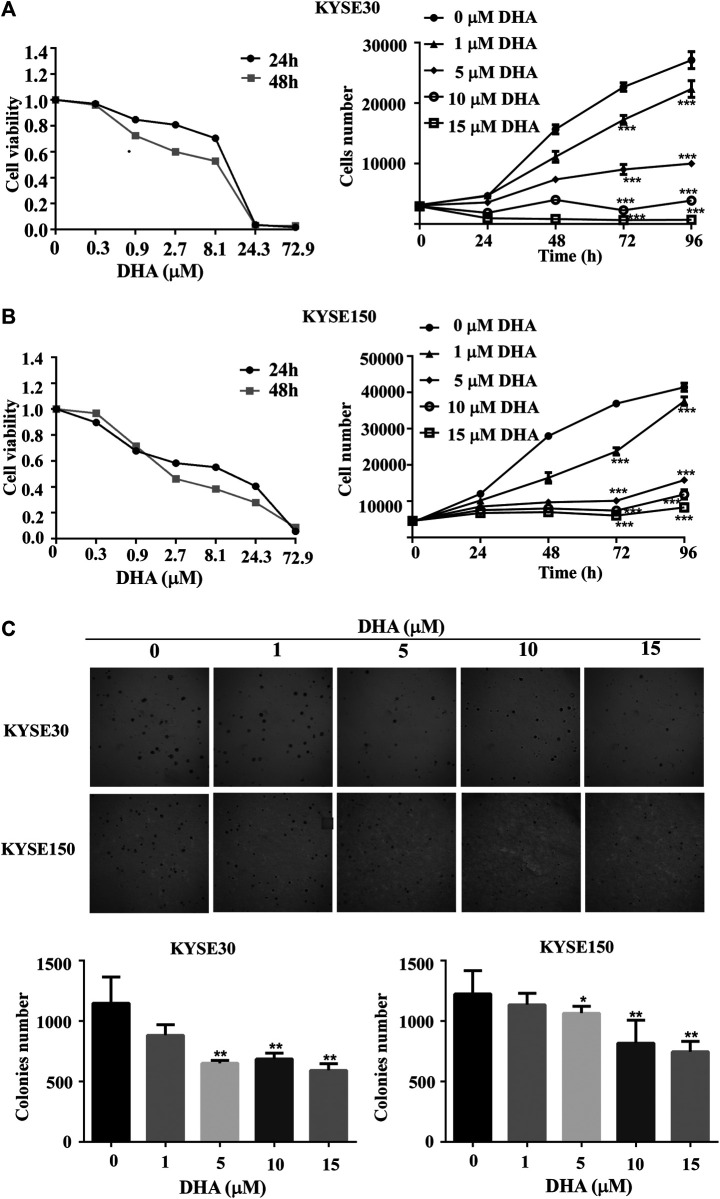
DHA inhibits proliferation and anchorage-independent growth of ESCC cells. **(A)** Toxicity (left panel) and growth inhibition (right panel) of DHA on KYSE30 cells. The number of cells was assessed by IN Cell Analyzer 6,000. Data are presented as means ± SD. **(B)** Toxicity (left panel) and growth inhibition (right panel) of DHA on KYSE150 cells. **(C)** DHA inhibited the colony-forming ability of ESCC cells. KYSE30 and KYSE150 cells were treated with DHA (0, 1, 5, 10 and 15 μM) in agar gel about 8 days respectively and the representative images are shown. The colony numbers of KYSE30 and KYSE150 were measured via In Cell 6,000 Analyzer. Data are presented as means ± SD (*, *p* < 0.05; **, *p* < 0.01; ***, *p* < 0.001) compared with the control group, *n* = 3 per group,one-way ANOVA).

To explore the effect of DHA on ESCC cells, KYSE30 and KYSE150 were treated with various doses of DHA. Through DHA cytotoxicity assay, we chose 0, 1, 5, 10, or 15 μM for subsequent experiments. As the concentration increased, DHA significantly inhibited the growth of KYSE30 (Figure 1A) and KYSE150 cell proliferation (Figure 1B). Anchorage-independent cell growth assay was used to further evaluate the antiproliferative effect of DHA. The colony size and colony number of KYSE30 and KYSE150 cells in those treated with DHA were lower than with controls (Figure 1C). These results indicated that DHA suppressed cells proliferative activity of ESCC *in vitro*.

**FIGURE 2:**
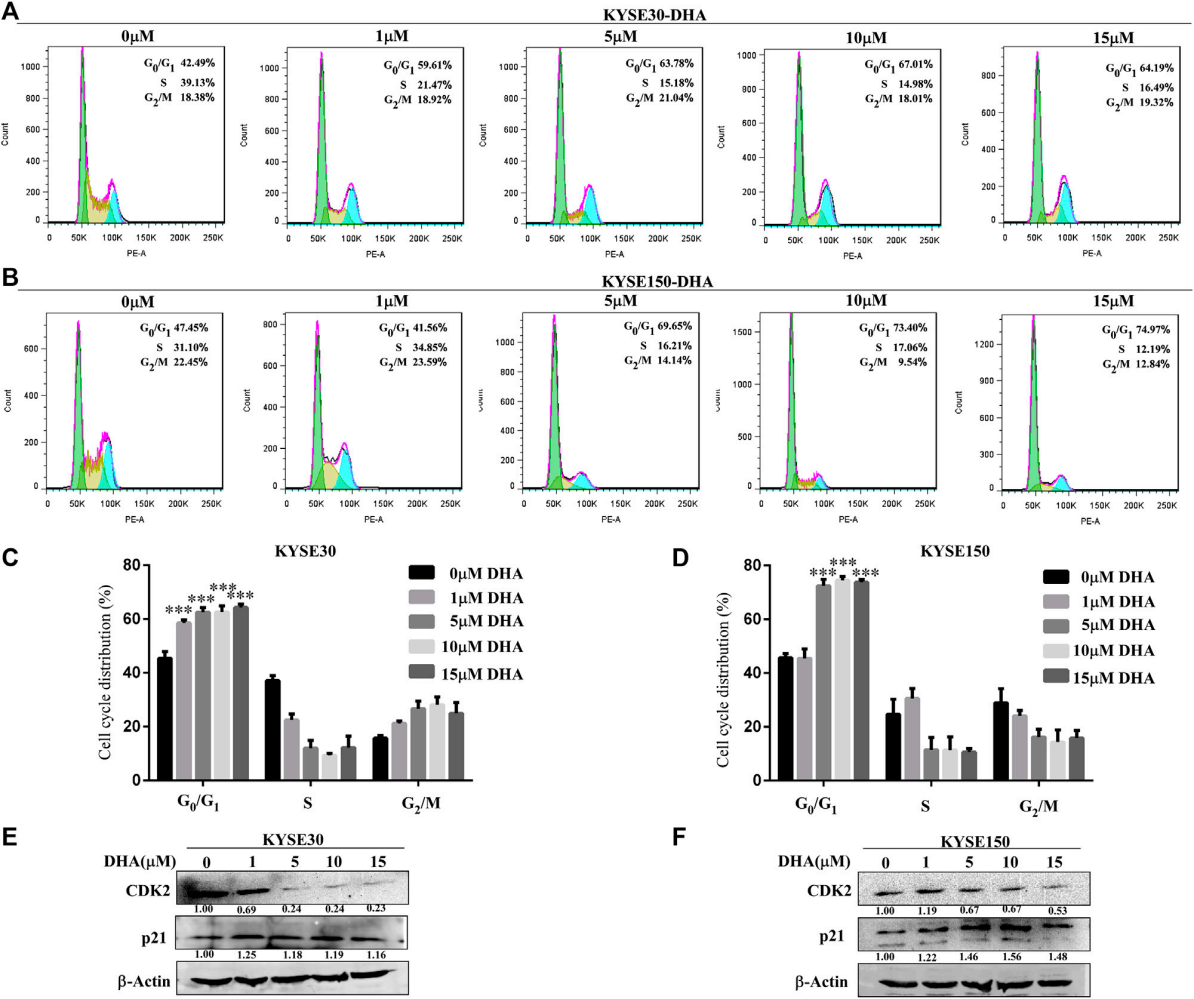
Effects of DHA on the cell cycle progression in KYSE30 and KYSE150 cells. **(A, B)** KYSE30 and KYSE150 cells were stained with propidium iodide (PI) and the cell cycle distribution was analyzed using flow cytometry after DHA treatment for 24 h. **(C)** The quantitative cell cycle distribution data in KYSE30 cells are shown **(D)** The quantitative cell cycle distribution data in KYSE150 cells are shown. Data are presented as mean ± SD (*, *p* < 0.05; **, *p* < 0.01; ***, *p* < 0.001; *n* = 3 per group, one-way ANOVA). **(E)** DHA affected the protein levels of the cell cycle-related CDK2 and p21 in KYSE30 cells **(F)** DHA affected the protein levels of the cell cycle-related CDK2 and p21 in KYSE150 cells.

We examined the effect of DHA on cell cycle progression in KYSE30 and KYSE150 cells for 24 h using propidium iodide staining. With the increase of DHA concentration, the cell cycles of KYSE30 (Figures 2A,C) and KYSE150 (Figures 2B,D) were significantly blocked in *G*_0_/*G*_1_ phase. To further confirm the effect of DHA on the cycle, we examined the protein levels of cyclin-dependent kinase 2 (CDK2) and p21 using western blot analysis. DHA attenuated the expression of CDK2 in KYSE30 (Figure 2E) and KYSE150 cells (Figure 2F). Protein p21 was upregulated by DHA in KYSE30 (Figure 2E) and KYSE150 cells (Figure 2F). These results suggested that DHA treatment affected cell cycle transition from *G*_0_/*G*_1_ to *S* phase.

### Dihydroartemisinin Inhibits Esophageal Squamous Cell Carcinoma Cell Proliferation by Attenuating mTOR-p70S6K-RPS6 Axis

**FIGURE 3:**
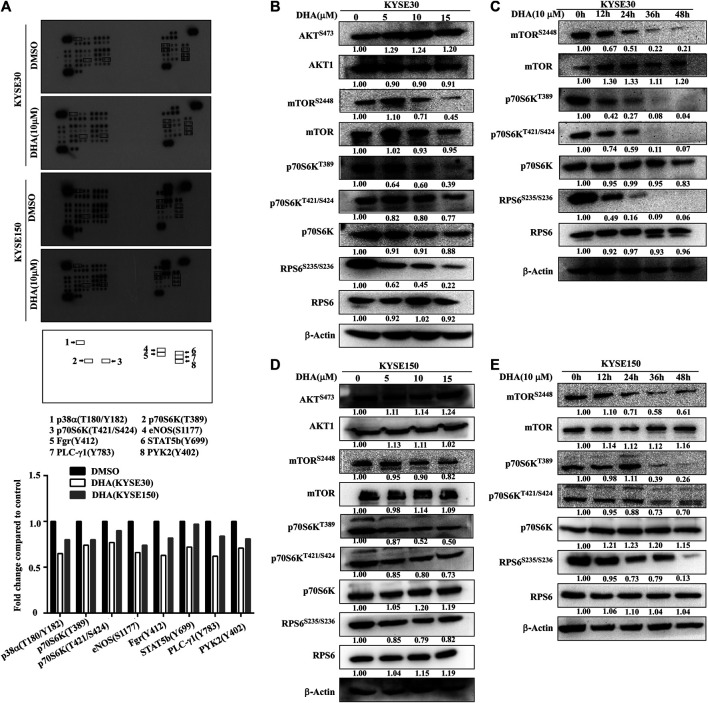
DHA attenuats phosphorylation levels of mTOR-p70S6K-RPS6. **(A)** Phospho-protein array analysis. Array spots were visualized using an ECL kit (above). The graph shows the fold change. Data are shown as an average of duplicate samples (below). **(B)** With the increasing concentration of DHA in KYSE30 cells (0, 5, 10, or 15 μM), the levels of p-mTOR^S2448^, p-p70S6K^T389^, p-p70S6K^T421/S424^ and p- RPS6^S235/S236^ were decreased, confirmed using western blot analysis. **(C)** As the time of DHA treatment in KYSE30 cells (0, 12, 24, 36, or 48 h) increased, the levels of p-mTOR^S2448^, p-p70S6K^T389^, p-p70S6K^T421/S424^, and p- RPS6^S235/S236^ also decreased, confirmed using western blot analysis. **(D)** With the increasing concentration of DHA in KYSE150 cells (0, 5, 10, or 15 μM), the levels of p-mTOR^S2448^, p-p70S6K^T389^, p-p70S6K^T421/S424^, and p- RPS6^S235/S236^ decreased, confirmed using western blot analysis. **(E)** As the time of DHA treatment in KYSE150 cells (0, 12, 24, 36, or 48 h) increased, the levels of p-mTOR^S2448^, p-p70S6K^T389^, p-p70S6K^T421/S424^ and p- RPS6^S235/S236^ decreased, confirmed using western blot analysis. Densitometry analyses were performed for all the western blot analyses using by ImageJ.

To analyze the effect of DHA on the phosphorylation protein of ESCC better, a protein kinase array was used to screen potential protein molecules. KYSE30 and KYSE150 cells were treated with DHA (10 μM) for 12 h. The results indicated that DHA inhibited the phosphorylation of p38α, p70S6K, eNOS, Fgr, STAT5b, PLC-*γ*1 and PYK2 (Figure 3A). Among them, the levels of 70-kDa ribosomal protein S6 kinases (p70S6K^T389^ and p70S6K^T421/S424^) were suppressed. Moreover, we explored the molecular pathways by which DHA affected mTOR signaling. We found that DHA weakened mTORC1 signaling by reducing the levels of phosphorylated mTOR at Ser2448 (p-mTOR^S2448^), p70S6K at Thr389 (p-p70S6K^T389^), p70S6K at Thr421/Ser424 (p-p70S6K^T421/S424^), and RPS6 at Ser235/Ser236 (p-RPS6^S235/S236^). Treatment with DHA at 0, 5, 10, or 15 μM for 24 h downregulated the levels of p-mTOR^S2448^, p-p70S6K^T389^, p-p70S6K^T421/S424^ and p-RPS6^S235/S236^ in KYSE30 cells (Figure 3B). Furthermore, 10 μM DHA-treated KYSE30 cells for 0, 12, 24, 36, or 48 h also showed a decrease in the phosphorylated proteins, but the total protein did not change (Figure 3C). Similarly, the DHA-treated KYSE150 cells had the same trend regardless of the concentration or incubation time (Figures 3D,E).

### Dihydroartemisinin Targets AKT1 and p70S6K to Affect mTOR Signaling Pathway in Esophageal Cancer

**FIGURE 4:**
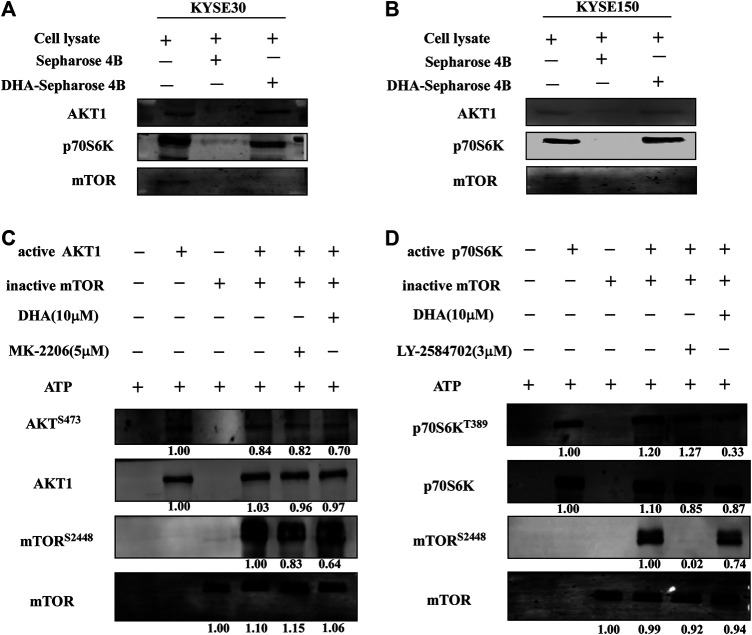
DHA affects mTOR signaling pathway in ESCC cells by targeting AKT1 and p70S6K. **(A)** DHA directly bound to AKT1 and p70S6K in KYSE30 cells **(B)** DHA directly bound to AKT1 and p70S6K in KYSE150 cells. ESCC cell lysates (500 μg) were incubated with DHA-conjugated Sepharose 4B beads or Sepharose 4B beads alone. The pulled-down proteins were analyzed using western blot analysis. **(C)** DHA attenuated the phosphorylation of mTOR by inhibiting the kinase activity of AKT1 **(D)** DHA attenuated the phosphorylation of mTOR by inhibiting the kinase activity of p70S6K.

From above results, it could be inferred that DHA inhibited the proliferation of ESCC cells by acting on the mTOR cascade signaling pathway. However, it was not clear as to which protein DHA specifically targeted. We first found that DHA did not bind to mTOR protein through *in vitro* pull-down assays (Figures 4A,B). However, DHA could bind to AKT1 and p70S6K, which proteins were extracted from KYSE30 and KYSE150 cells (Figures 4A,B). We once again confirmed that AKT1 and p70S6K directly phosphorylated S2448 site of mTOR through an *in vitro* kinase assay, and DHA significantly attenuated the phosphorylation level of mTOR through AKT1 and p70S6K (Figures 4C,D). What’s more, MK2206 (an AKT inhibitor) and LY-2584702 (a p70S6K inhibitor) reduced the phosphorylation of mTOR in the *in vitro* kinase assays and in ESCC cells, respectively (Figures 4C,D; Supplementary Figure S1). These results suggested that AKT1 and p70S6K involved in the phosphorylation of mTOR jointly and DHA-induced inhibition of mTOR might be due to affecting the kinases activity of AKT1 and p70S6K partially.

### Dihydroartemisinin Inhibits Patient-Derived Xenograft Esophageal Squamous Cell Carcinoma Tumor Growth *in vivo*

**FIGURE 5:**
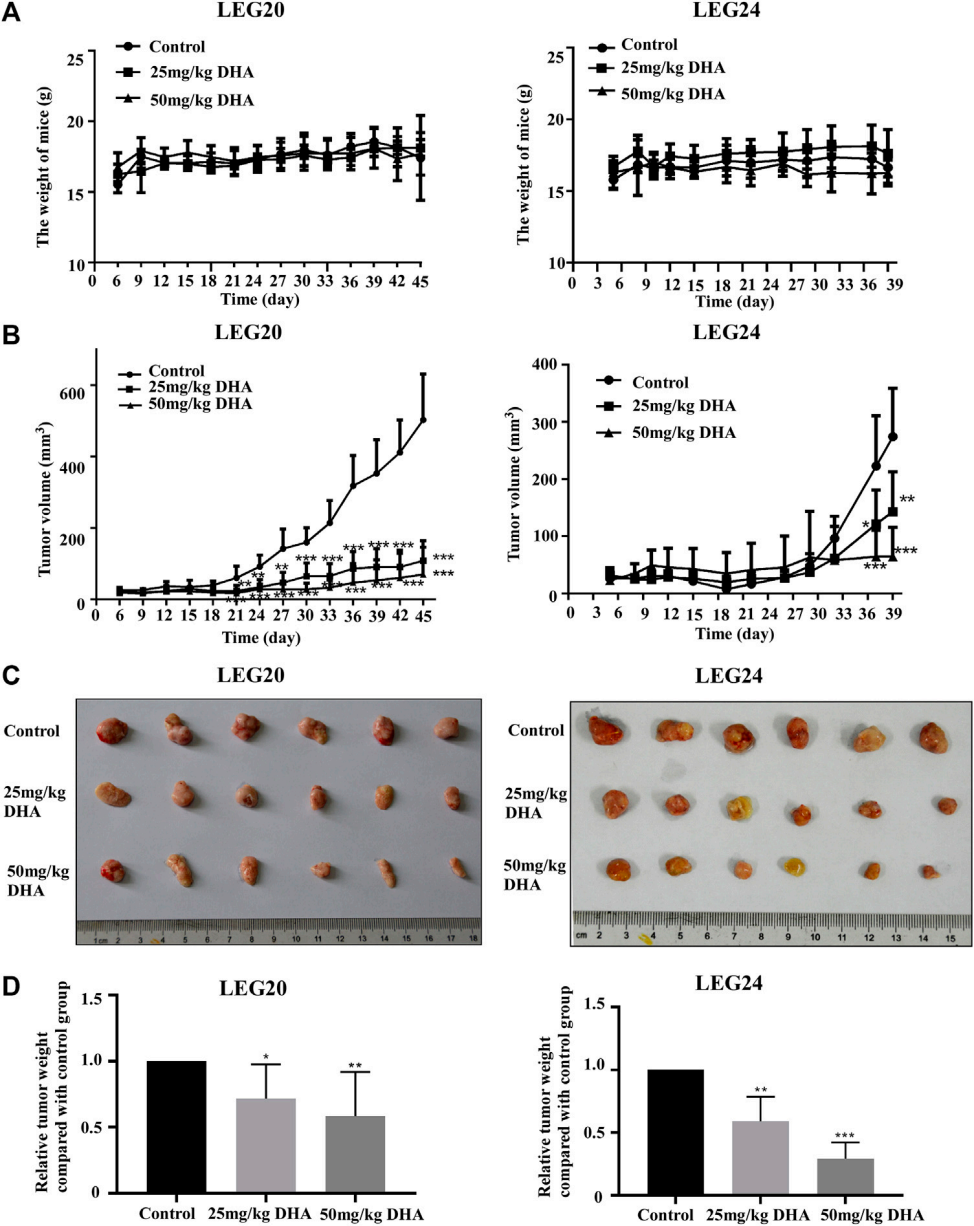
DHA suppresses tumor growth (LEG20 and LEG24) of ESCC *in vivo*. **(A)** DHA did not affect the body weight of the mice **(B)** DHA inhibited the growth of xenograft tumors. Tumor volume in each mouse was measured using a caliper once every 3 days. **(C)** When the mice were sacrificed, the tumors were extracted and photographed **(D)** Tumor weights were decreased in the DHA treatment group, confirmed by analyzing the weight of tumors in each group (*, *p* < 0.05; **, *p* < 0.01; ***, *p* < 0.001; *n* = 6 per group, one-way ANOVA).

To confirm the inhibitory effect of DHA *in vivo*, we selected two patient cases (LEG20 and LEG24) as PDX models for further studies. The PDX tumor tissues were implanted into the back of the neck of SCID mice; either control or DHA (25 or 50 mg/kg body weight) was administered through intraperitoneal injection every 2 days. The results showed that DHA significantly inhibited tumor growth compared with the control group (Figure 5C). With administrate time extended, the tumor of mice treated with 25 and 50 mg/kg of DHA grew slower than that of control group (Figure 5B). Tumor tissues were weighed after the mice were euthanized. The weights of the tumor tissues treated with DHA (Figure 5D) were lower than those of the control group. As the concentration of DHA increased, the volume and weight of tumors decreased. In these two cases, DHA significantly inhibited tumor growth without loss in body weight with reference to the control group (Figure 5A).

### Dihydroartemisinin Inhibits p-mTOR, p-p70S6K, p-RPS6 Levels in Tumor Tissues

**FIGURE 6:**
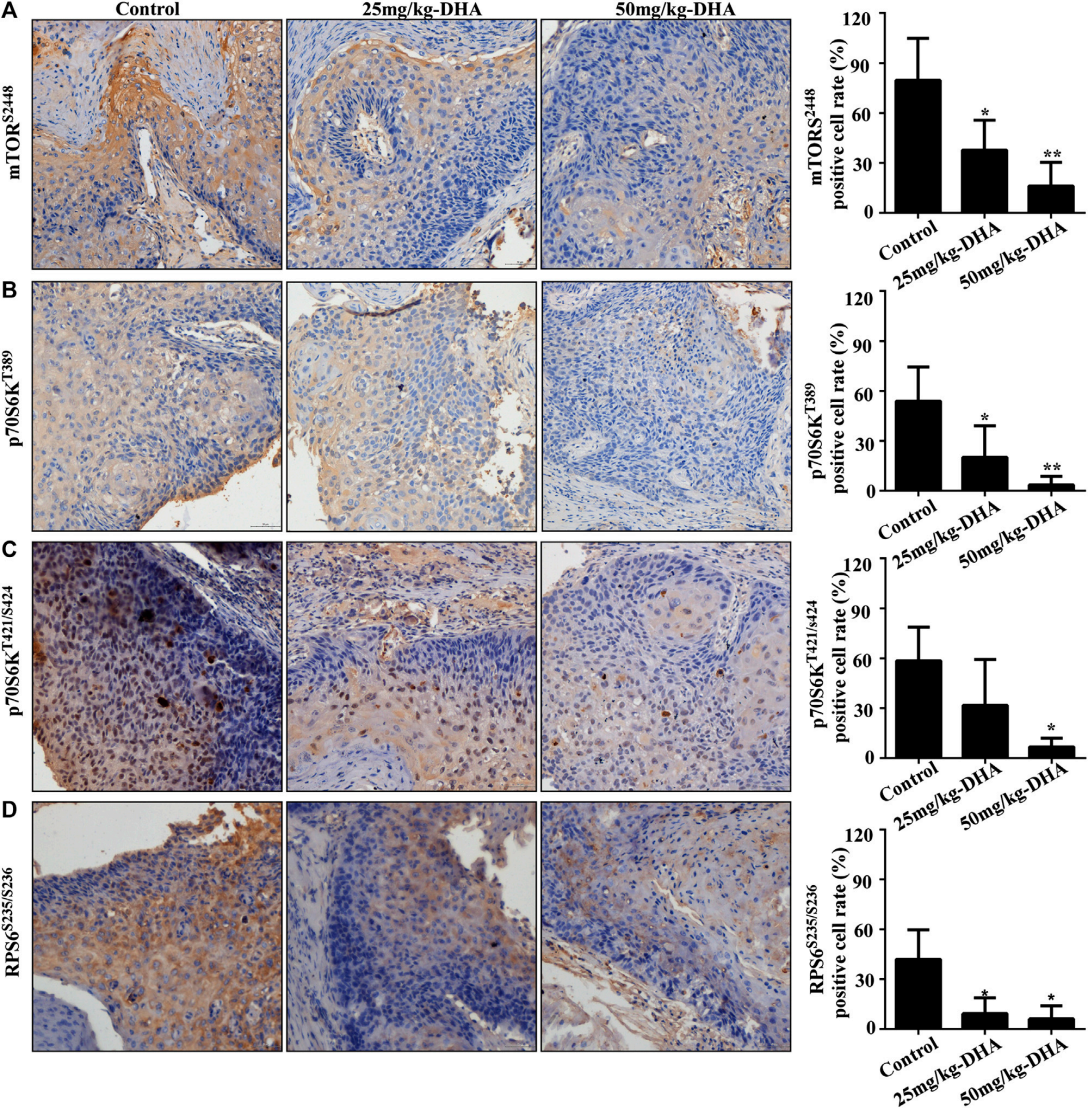
DHA suppresses the phosphorylation of mTOR^S2448^, p70S6K^T389^, p70S6K^T421/S424^, and RPS6^S235/S236^ in the LEG20 xenograft mice model, confirmed using IHC analysis. **(A–D)** DHA inhibited the levels of mTOR^S2448^ **(A)** p70S6K^T389^ **(B)** p70S6K^T421/S424^ **(C)** and RPS6^S235/S236^ **(D)** in the LEG20 xenograft tumors. Representative images of each group are shown. All the sections were scanned using TissueFAXS (400×), and the positive cells were analyzed using HistoQuest 4.0 software (*, *p* < 0.05; **, *p* < 0.01; ***, *p* < 0.001; *n* = 6 per group, one-way ANOVA).

To determine whether mTOR pathway proteins were involved in the enhanced therapeutic effects exerted by DHA, we examined the changes in phosphorylated protein levels by comparing PDX samples from LEG20 and LEG24 cases treated with the control or DHA (25 mg/kg and 50 mg/kg). We investigated the effect of DHA on p-mTOR^S2448^, p-p70S6K^T389^, p-p70S6K^T421/S424^ and p-RPS6^S235/S236^ by immunohistochemical (IHC) analysis of PDX LEG20 and LEG24 tumor samples. The phosphorylation of mTOR, p70S6K, RPS6 were strongly inhibited in the DHA-treated group (Figure 6; Supplementary Figure S3), which also proved by western blot analysis (Supplementary Figure S2). These results suggested that DHA suppressed the growth of patient-derived esophageal tumor through mTOR-p70S6K pathway *in vivo*.

## Discussion

In this study, we showed that DHA inhibited the proliferation of ESCC *in vitro* and *in vivo* via the AKT1-mTOR-p70S6K axis. Esophageal cancer is one of the most common malignant tumors in the world. Despite aggressive surgical resection and chemoradiation, its prognosis is still poor. There is an urgent need to identify new drugs capable of inhibiting ESCC progression. Chinese herbal medicine is an important resource for discovering new medicines, and natural products derived from herbal medicines have significant anticancer activity (Luo et al., 2019). As an effective antimalarial substance, DHA has attracted widespread attention for its anticancer effect and low toxicity.

Previous studies showed that DHA inhibited cell proliferation and induced apoptosis in human hepatocellular carcinoma cells by upregulating TNF expression via JNK/NF-κB pathways (Wu et al., 2019). Li et al. demonstrates that DHA may repress esophageal cancer glycolysis partly by down-regulating PKM2 (Li et al., 2019). Although the above studies show the inhibitory effect of DHA on tumor cells, they are all *in vitro* experiments, and the molecular mechanism is not detailed. Here, with the increase of DHA concentration, the growth and colony forming ability of ESCC were significantly inhibited (Figure 1). DHA also significantly inhibited the growth and reduced the size and weight of the two cases of ESCC tumors *in vivo* (Figure 5). Furthermore, DHA induced cell cycle arrest in G_1_ phase, and the related regulatory proteins CDK2 and p21 changed correspondingly (Figure 2). CDK2 is a key regulator of cell cycle transitions and controls the G_1_ to S phase transition (Ekholm and Reed, 2000). Decreased levels of CDK2 cause typical cell cycle arrest at the *G*_1_/*S* phase (Lian et al., 2019). Moreover, p21 can arrest the cell cycle by inhibiting CDKs (Abbas and Dutta, 2009). Multiple studies have suggested that inhibiting the activity of mTOR blocks the cycle of cancer cells and further inhibits proliferation (Chen et al., 2018; Phyu and Smith, 2019). In our study, we proved that DHA inhibited the mTOR pathway (Figure 3). Therefore, we speculated that the cell cycle arrest of ESCC induced by DHA was related to the inhibition of mTOR pathway.

Evidence suggests that DHA can affect the AKT and/or mTOR pathway in different diseases. DHA treatment inhibits the phosphorylation of AKT and induces glioma cells apoptosis (Du et al., 2015; Shao et al., 2017). In the autoimmune thyroiditis (AIT), DHA inhibits the CXCR3/PI3K/AKT/NF-κB signaling pathway which executes important roles in AIT (Liu et al., 2017). DHA and gefitinib co-administration also downregulate the levels of phosphorylated p-AKT and p-mTOR to inhibit the proliferation and migration of non-small cell lung cancer cells (Jin et al., 2017). Recent studies indicate that DHA induces cell autophagy via mTOR/p70S6k signaling pathway (Du et al., 2019; Liu et al., 2019). Therefore it is considered that the inhibitory effect of DHA on cells is multi-target and multi-pathway. In our study, DHA did not significantly reduce the phosphorylation of AKT in ESCC cells, but significantly inhibited the phosphorylation of mTOR (Figures 3B,D). Next, We found that DHA affected the mTOR-p70S6K-RPS6 signaling pathway in ESCC cells (Figure 3). However, the direct target protein of DHA remains unknown. The results of the pull-down assay indicated that DHA did not bind the mTOR (Figures 4A,B). Studies have shown that AKT and p70S6K phosphorylate the S2448 site of mTOR protein to activate the mTOR pathway (Nave et al., 1999; Chiang and Abraham, 2005). Chiang and Abraham also proved that p70S6 kinase phosphorylates mTOR at Ser-2448 *in vitro*, and that ectopic expression of p70S6 kinase restores Ser-2448 phosphorylation in rapamycin-treated cells. These reports, combined with our results suggested that DHA may inhibit the mTOR pathway through affecting AKT or p70S6K. Next, our results demonstrated that active AKT1 (S473) and p70S6K (T389) phosphorylated S2448 site of mTOR in an *in vitro* kinase assay. Moreover, DHA inhibited phosphorylation of mTOR by binding to AKT1 and p70S6K (Figures 4C,D). To further confirm these phenomena, we measured the mTOR phosphorylation levels in kinase inhibitor (MK2206 and LY-2584702)-treated ESCC cells. The results showed that the inhibition of AKT and p70S6K kinase activity weakened mTOR phosphorylation (Figures 4C,D; Supplementary Figure S1). Likewise, MK2206 and LY-2584702 decreased the phosphorylation level of mTOR via AKT-S473 and p70S6K-T389 in the *in vitro* kinase assay. IHC analysis and western blot analyses revealed that the levels of p-mTOR, p-p70S6K, and p-RPS6 in tumor tissues were inhibited by DHA *in vivo* (Figure 6; Supplementary Figures S2 and S3). Thus, DHA was consided as a potential inhibitor of the mTOR-p70S6K-RPS6 pathway partially by affecting the kinase activity of AKT1 and p70S6K (Supplementary Figure S4).

In conclusion, our study suggests that DHA inhibits the mTOR pathway partially by targeting AKT1 and p70S6K, which attenuates the proliferation of ESCC *in vitro* and *in vivo*.

## Data Availability Statement

The original contributions presented in the study are included in the article/Supplementary Material, further inquiries can be directed to the corresponding author/s.

## Ethics Statement

The study was approved and supervised by the research ethics committee of Zhengzhou University, Zhengzhou, China. All animal experiments were carried out in accordance with the guidelines for animal experiments at Zhengzhou University.

## Author Contributions

JMZ, XHC, and LLZ designed the study. LLZ, YYZ, JCQ, TTN, YWD, and YX performed the experiments. JMZ, XHC, and LLZ analyzed the data and wrote the manuscript. YNJ, KDL, JL, WJY, YQ, GJ, JFM, XHC, and ZMD reviewed and edited the manuscript. All authors read and approved the final manuscript.

## Funding

This study was supported by the National Natural Science Foundation of China (Grant no. 81472324, 81872335, and 81702380), the Science Foundation of Henan Education Department (Grant no. 20A310023 and 19A310026) and the Supporting Plan of Scientific and Technological Innovation Team in Universities of Henan Province (No. 20IRTSTHN029).

## Conflict of Interest

The authors declare that the research was conducted in the absence of any commercial or financial relationships that could be construed as a potential conflict of interest.

## Abbreviations

DHA, dihydroartemisinin; ESCC, esophageal squamous cell carcinoma; SCID, the severe combined immunodeficient mice; PDX, patient derived xenograft; IHC, immunohistochemical staining
