# Supplementary material for: Dihydroartemisinin Inhibits the Proliferation of Esophageal Squamous Cell Carcinoma Partially by Targeting AKT1 and p70S6K
Source: Front Pharmacol. 2020 Nov 20;11:587470. doi: 10.3389/fphar.2020.587470 (PMC7919191; doi:10.3389/fphar.2020.587470)
Supplement: Supplementary file 1 [file DataSheet1.pdf]

Supplementary material

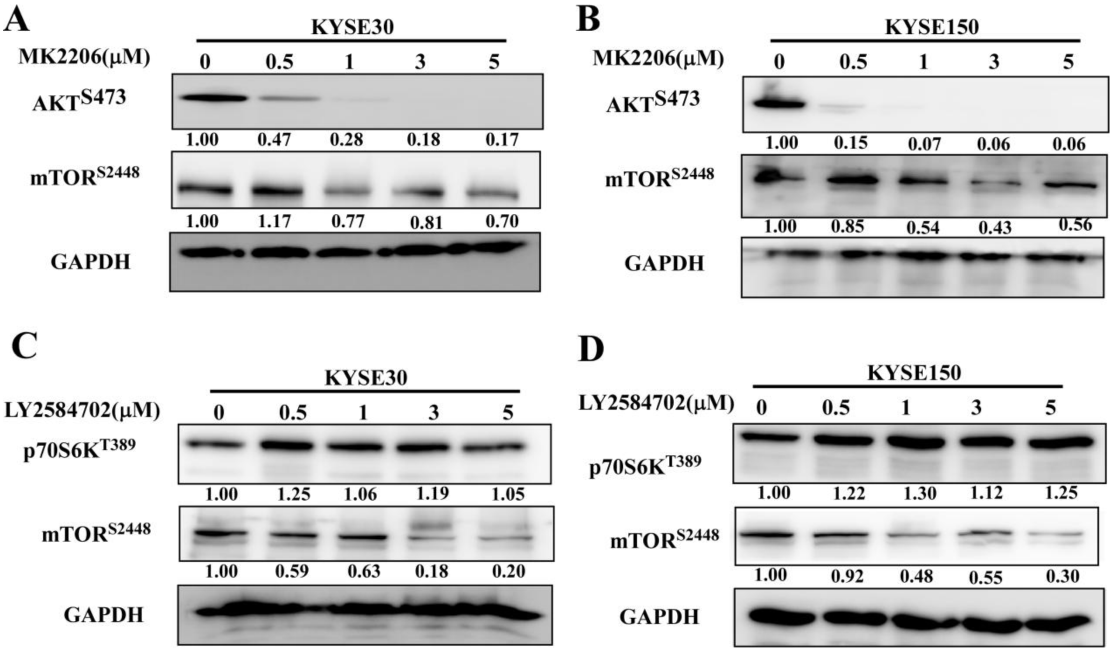

Supplementary Figure1. MK2206 and LY-2584702 reduced the phosphorylation level of mTOR respectively. **(A-B)** The phosphorylation level of mTOR of KYSE30 or KYSE150 cells was weakened by MK2206 treatment for 4 h. **(C-D)** The phosphorylation level of mTOR of KYSE30 or KYSE150 cells was weakened by LY-2584702 treatment for 4 h. Densitometry analyses were performed for all the western blots by Image J.

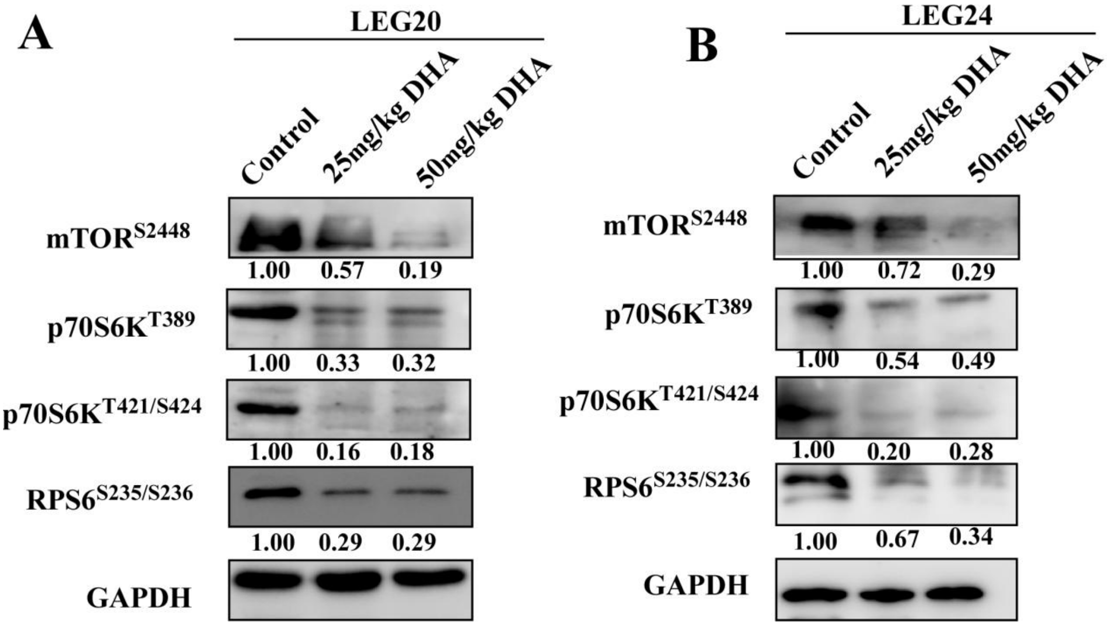

Supplementary Figure2. DHA suppresses the phosphorylation of mTOR^{S2448}, p70S6K^{T389}, p70S6K$^{T421/S424}$ and RPS6$^{S235/S236}$ of tumor tissues in xenograft mice model using western blot analysis. **(A)** LEG20 case. **(B)** LEG24 case. Densitometry analyses were performed for all the western blots by Image J.

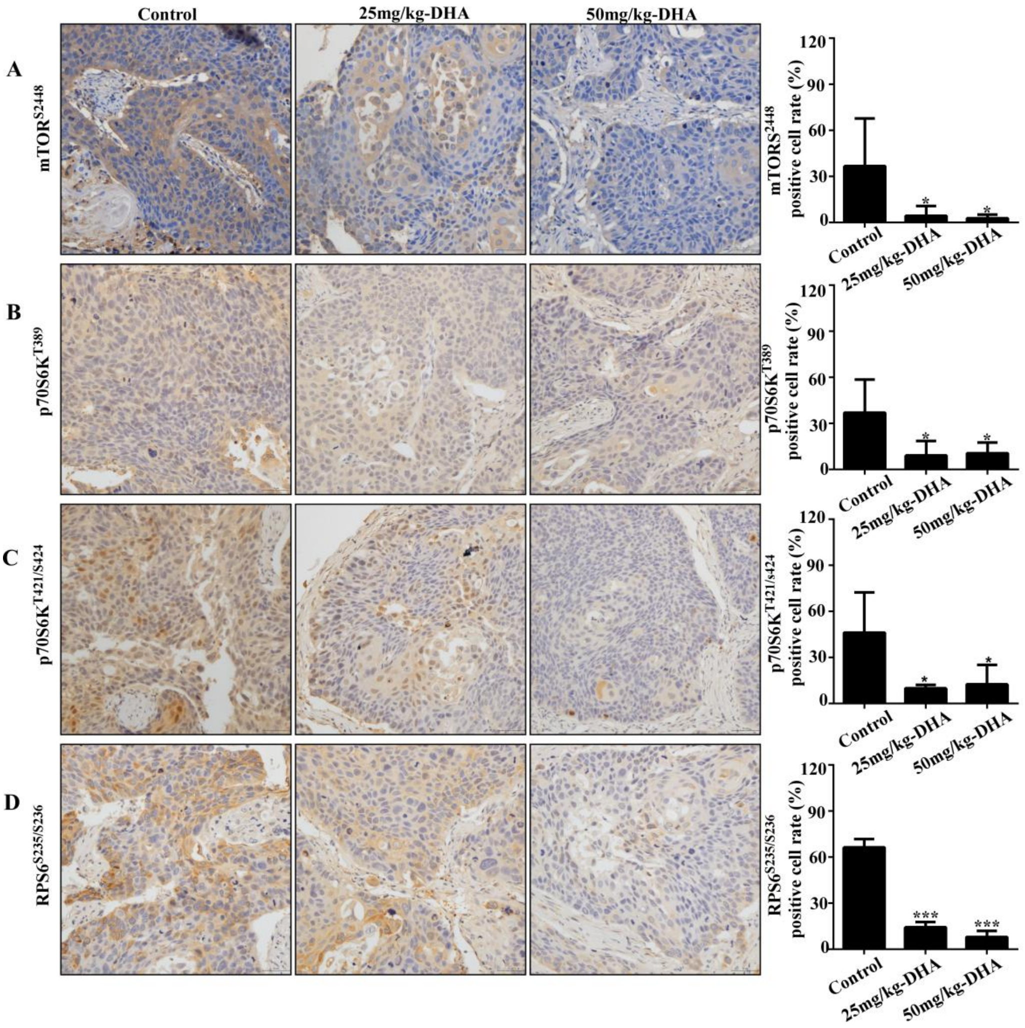

Supplementary Figure3. DHA suppresses the phosphorylation of mTOR^{S2448}, p70S6K^{T389}, p70S6K$^{T421/S424}$, and RPS6$^{S235/S236}$ in the LEG24 xenograft mice model, confirmed using IHC analysis. **(A-D)** DHA inhibited the levels of mTOR^{S2448}**(A),** p70S6K^{T389}**(B)**, p70S6K$^{T421/S424}$**(C)** and RPS6$^{S235/S236}$**(D)** in the LEG24 xenograft tumors. Representative images of each group are shown. All the sections were scanned by using TissueFAXS (400×), and the positive cells were analyzed using HistoQuest 4.0 software.(*, $p < 0.05$; **, $p < 0.01$; ***, $p < 0.001$; n = 6 per group, one-way ANOVA)

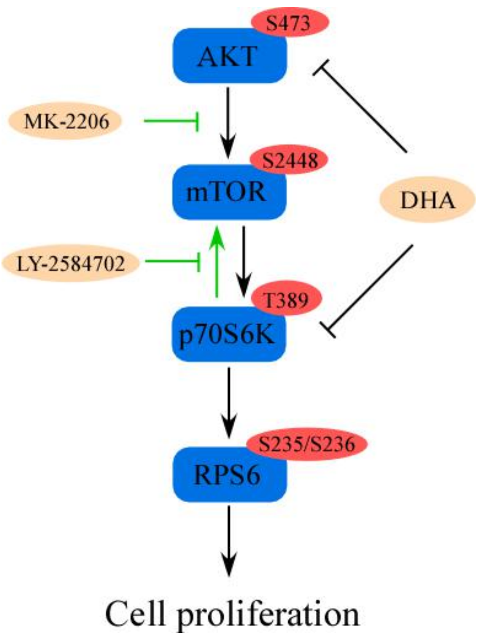

Supplementary Figure 4. The graphic summary that DHA inhibits ESCC cell proliferation via AKT1-mTOR-p70S6K axis.
